# Next-generation sequencing yields the complete mitogenome of *Caridina multidentata* and phylogenetic analysis

**DOI:** 10.1080/23802359.2017.1422403

**Published:** 2018-01-03

**Authors:** Zhengfei Wang, Qiong Wu, Huayun Guo, Dan Tang, Yuze Bai, Ziqian Wang, Yitao Tao

**Affiliations:** Jiangsu Key Laboratory for Bioresources of Saline Soils, Jiangsu Synthetic Innovation Center for Coastal Bio-agriculture, Jiangsu Provincial Key Laboratory of Coastal Wetland Bioresources and Environmental Protection, School of Ocean and Biological Engineering, Yancheng Teachers University, Yancheng, Jiangsu Province, China

**Keywords:** Mitogenome, phylogentic, caridea, atyidae, *Caridina multidentata*

## Abstract

In this study, the complete mitochondrial genome (mitogenome) of *Caridina multidentata* is reported for the first time. These data demonstrate that the *C. multidentata* mitochondrial genome is a 15,825 bp circular molecule and encodes the typical 37 metazoan mitochondrial genes [13 protein-coding genes (PCGs), 22 transfer RNA genes (tRNAs), two ribosomal RNA genes (rRNAs)] and an A + T-rich control region. The genome composition was highly A + T biased 63.45% and showed negative AT-skew (−0.131) and slightly possitive GC-skew (0.018). The complete mitogenome provides essential and important DNA molecular data for further phylogenetic and evolutionary analysis for shrimps.

## Introduction

Mitogenome sequences are commonly utilized as genetic markers in molecular genetic studies for decapoda crustacean species. So far, the Caridea are represented by only 17 complete mitogenomes on the NCBI public database (Tan et al. 2017). The complete mitogenome of *C. multidentata* (Crustacea, Decapoda, Atyidae) has not been reported and the phylogenetic position of Caridea within decapods has not been settled, although there is good evidence that they belong to the large group Pleocyemata (Burkenroad 1963; Tsang et al. 2008; Klann and Scholtz 2014). In this study, we determined the complete mitogenome of *C. multidentata* and constructed phylogenetic trees to provide insight into phylogenetic relationships of *C. multidentata* and related species.

Samples of *C. multidentata* were collected from the coastal waters off Keelung, Taiwan, China (25°09′23″ N 121°44′10″ E). All samples were stored at the Jiangsu Provincial Key Laboratory of Coastal Wetland Bioresources and Environmental Protection, Yancheng Teachers University, Yancheng, Jiangsu Province, China. Total DNA was extracted from the muscle tissues and using the Aidlab Genomic DNA Extraction Kit (Aidlab Biotech, Beijing, China). The mitogenomes of *C. multidentata* were sequenced by next-generation sequencing (Illumina HisSeq 4000; Shanghai Origingene Bio-pharm Technology Co. Ltd., Shanghai, China). Raw sequence data (4 Gb rawdata) were deposited into Short Read Archive (SRA) database (https://www.ncbi.nlm.nih.gov/sra/) with the accession no. SRR6315541. Clean data without sequencing adapters were *de novo* assembled using the NOVO Plasty software (Dierckxsens et al. 2017). Average sequencing depth of ∼78 × was obtained for the mitogenome.

The mitogenome of *C. multidentata* is a closed circular molecule 15,825 bp in size and encodes the 37 genes [13 PCGs (*cox1-3*, *nad1-6*, *nad4L*, *cob*, *atp6* and *atp8*), 22 tRNAs (one for each amino acid, two for Leucine and Serine), two rRNAs (*rrnS* and *rrnL*) and a major non-coding region known as the CR]. The nucleotide composition of the complete mitogenome is as follows: A = 27.57%, T = 35.88%, G = 18.60% and C = 17.94%. The whole mitogenome of *C. multidentata* was biased toward AT nucleotides (63.45%). The AT-skew and GC-skew for the whole mitogenome are negative and positive, respectively, indicating a higher occurrence of T than A and G than C. The entire *C. multidentata* mitochondrial genome sequence has been deposited in GenBank with accession number MG580781.

**Figure 1.**
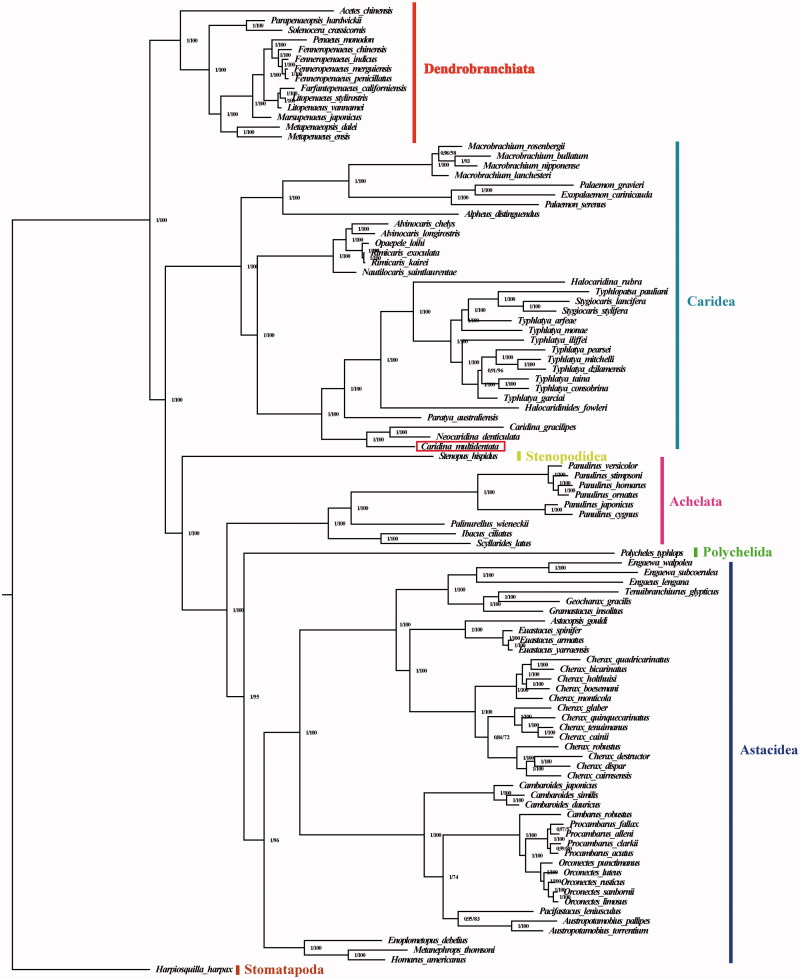
Phylogeny of shrimps based on amino acid sequences of 13 mitogenome PCGs using BI and ML methods. Numbers on branches indicate posterior probability (BI) and bootstrap (ML). *Harpiosquilla harpax* was used as the outgroup. The gene’s accession number for tree construction is listed as follows: *Acetes chinensis* (NC_017600), *Parapenaeopsis hardwickii* (NC_030277), *Solenocera crassicornis* (NC_030280), *Marsupenaeus japonicus* (NC_007010), *Farfantepenaeus californiensis* (NC_012738), *Litopenaeus vannamei* (NC_009626), *Litopenaeus stylirostris* (NC_012060), *Penaeus monodon* (NC_002184), *Fenneropenaeus chinensis* (NC_009679), *Fenneropenaeus indicus* (NC_031366), *Fenneropenaeus merguiensis* (NC_026884), *Fenneropenaeus penicillatus* (NC_026885), *Metapenaeus ensis* (NC_026834), *Metapenaeopsis dalei* (NC_029457), *Alpheus distinguendus* (NC_014883), *Palaemon serenus* (NC_027601), *Exopalaemon carinicauda* (NC_012566), *Palaemon gravieri* (NC_029240), *Macrobrachium rosenbergii* (NC_006880), *Macrobrachium lanchesteri* (NC_012217), *Macrobrachium bullatum* (NC_027602), *Macrobrachium nipponense* (NC_015073), *Nautilocaris saintlaurentae* (NC_021971), *Alvinocaris chelys* (NC_018778), *Alvinocaris longirostris* (NC_020313), *Opaepele loihi* (NC_020311), *Rimicaris exoculata* (NC_027116), *Rimicaris kairei* (NC_020310), *Halocaridinides fowleri* (NC_035412), *Halocaridina rubra* (NC_008413), *Typhlopatsa pauliani* (NC_035406), *Stygiocaris lancifera* (NC_035404), *Stygiocaris stylifera* (NC_035411), *Typhlatya monae* (NC_035405), *Typhlatya arfeae* (NC_035410), *Typhlatya iliffei* (NC_035401), *Typhlatya pearsei* (NC_035400), *Typhlatya mitchelli* (NC_035403), *Typhlatya dzilamensis* (NC_035408), *Typhlatya garciai* (NC_035409), *Typhlatya consobrina* (NC_035407), *Typhlatya taina* (NC_035399), *Paratya australiensis* (NC_027603), *Neocaridina denticulata* (NC_023823), *Caridina gracilipes* (NC_024751), *Stenopus hispidus* (NC_018097), *Panulirus stimpsoni* (NC_014339), *Panulirus ornatus* (NC_014854), *Panulirus homarus* (NC_016015), *Panulirus versicolor* (NC_028627), *Panulirus japonicus* (NC_004251), *Panulirus cygnus* (NC_028024), *Palinurellus wieneckii* (NC_021753), *Scyllarides latus* (NC_020022), *Ibacus ciliatus* (NC_025581), *Polycheles typhlops* (NC_020026), *Astacopsis gouldi* (NC_026215), *Euastacus spinifer* (NC_026214), *Euastacus armatus* (NC_026575), *Euastacus yarraensis* (NC_023811), *Cherax quadricarinatus* (NC_022937), *Cherax bicarinatus* (NC_026226), *Cherax monticola* (NC_022938), *Cherax holthuisi* (NC_026227), *Cherax boesemani* (NC_026224), *Cherax glaber* (NC_022939), *Cherax quinquecarinatus* (NC_023479), *Cherax tenuimanus* (NC_026559), *Cherax cainii* (NC_022936), *Cherax destructor* (NC_011243), *Cherax dispar* (NC_023480), *Cherax cairnsensis* (NC_023481), *Cherax robustus* (NC_023478), *Engaeus lengana* (NC_022847), *Engaewa subcoerulea* (NC_029407), *Engaewa walpolea* (NC_029395), *Tenuibranchiurus glypticus* (NC_025647), *Geocharax gracilis* (NC_023810), *Gramastacus insolitus* (NC_030531), *Cambaroides japonicus* (NC_033506), *Cambaroides similis* (NC_016925), *Cambaroides dauricus* (NC_033505), *Cambarus robustus* (NC_033507), *Orconectes rusticus* (NC_029720), *Orconectes sanbornii* (NC_029721), *Orconectes limosus* (NC_026561), *Orconectes luteus* (NC_033508), *Orconectes punctimanus* (NC_030768), *Procambarus alleni* (NC_028447), *Procambarus fallax* (NC_020021), *Procambarus clarkii* (NC_016926), *Procambarus acutus* (NC_033510), *Pacifastacus leniusculus* (NC_033509), *Austropotamobius pallipes* (NC_026560), *Austropotamobius torrentium* (NC_033504), *Enoplometopus debelius* (NC_025592), *Metanephrops thomsoni* (NC_027608), *Homarus americanus* (NC_015607), *Harpiosquilla harpax* (NC_006916).

To reconstruct the phylogenetic relationship among shrimps, the concatenated set of amino acid sequences of the 13 PCGs from 99 shrimps and one outgroup (*Harpiosquilla harpax*) was used for reconstructing phylogenetic relationships among the shrimps by Bayesian inference (BI) and maximum-likelihood (ML) methods. The topologies of phylogenetic analysis in BI and ML were roughly the same (Figure 1). We found *C. multidentata* and (*C. gracilipes + Neocaridina denticulata*) were clustered in one branch with high nodal support value, suggesting this species to Atyidae within Infraorder Caridea. Additionally, the results of the phylogenetic analyses revealed six main infraorder which is coincident with previous studies: Dendrobranchiata + (Caridea + (Stenopodidea + (Achelata +(Astacidea + Polychelida)))); there was strong support for the six clades (Shen et al. 2013; Kenny et al. 2014; Wang et al. 2017).
